# The Syndrome We Agreed to Call Bronchiolitis

**DOI:** 10.1093/infdis/jiz082

**Published:** 2019-02-19

**Authors:** Fernando P Polack, Renato T Stein, Adnan Custovic

**Affiliations:** 1 Fundación Infant, Buenos Aires, Argentina; 2 Centro Infant at Pontificia Universidade Catolica de Rio Grande do Sul, Porto Alegre, Brasil; 3 Department of Paediatrics, Imperial College, London, United Kingdom

**Keywords:** respiratory syncytial virus, bronchiolitis, machine learning, endotypes

## Abstract

We are ignoring evidence suggesting that the diagnosis of bronchiolitis encompasses several diseases with distinct underlying mechanisms, considerable heterogeneity in treatment responses, and ultimately different therapeutic targets. Understanding this heterogeneity may be the only way to deliver appropriate, stratified treatments.

Routine administration of bronchodilators and systemic or inhaled corticosteroids to treat acute bronchiolitis is discouraged in expert guidelines, because a number of randomized controlled trials (RCTs) in infants and young children have failed to demonstrate reproducible benefits of these drugs over placebo [1]. Yet, most practicing pediatricians witness at least a handful of children every season who seem to improve considerably with these treatments. If indeed some infants with bronchiolitis benefit from treatments such as inhaled corticosteroids, then one explanation for the lack of benefit in RCTs is that the same treatment(s) may have harmful effects in another subgroup [2]. *Primum non nocere* remains an important ethical principle of pediatric practice, and to deliver it in the context of bronchiolitis, we will have to move from the current symptom-based, “trial-and-error” approach, to a stratified, mechanism-based therapy. These observations confront us with a challenge: Are we ignoring the evidence that the diagnosis of bronchiolitis encompasses several diseases with distinct underlying mechanisms, considerable heterogeneity in treatment responses, and ultimately different therapeutic targets? If so, understanding this heterogeneity may be the only way to deliver stratified treatments.

Bronchiolitis was described in 1941 as an inflammatory “respiratory obstruction caused by mucus in the bronchioles” presenting “with a slight temperature, pharyngeal cough and some gastrointestinal upset,” followed by a phase when “bronchioles become plugged with exudate and the clinical picture is dominated by obstructive dyspnea. Respiratory distress is then very marked ... Cough is always incessant and disturbing” [3]. Seventy-eight years later, little has changed in its definition. Bronchiolitis is now, according to the American Academy of Pediatrics (AAP), “a constellation of clinical symptoms and signs including a viral upper respiratory prodrome followed by increased respiratory effort and wheezing … characterized by acute inflammation, edema and necrosis of epithelial cells lining small airways, increased mucus production, and bronchospasm” [1]. AAP guidelines exclude recurrent wheeze from the definition. There is no clear scientific evidence today for treating recurrent symptoms, which may occur weeks or months after a first episode, differently than a first wheezing event. “Acute bronchiolitis” and its many associated terms remain a fuzzy syndrome, with many flavors under the same umbrella.

The clinical presentation of bronchiolitis is far from monolithic. Bronchiolitis may coexist with viral pneumonia, present with more or less air entrapment, wheezing, cough, or hyperreactivity, and a range from scarce to abundant production of secretions. These different observable characteristics (phenotypes) spurred a number of diverse mechanistic hypotheses, all supported and disputed by well-conducted studies over the years. This rationale includes innate inflammation, Th2-mediated bronchoconstriction, direct viral injury of the small airways, and airway plugging due to debris and mucus production [4]. The fact that different mechanistic studies report contradictory findings does not necessarily make any of them incorrect, but may be a consequence of the heterogeneity of the primary outcome—the “syndrome we agreed to call bronchiolitis” (SWAB).

SWAB can be caused by different viruses. The most frequent cause is respiratory syncytial virus (RSV), associated with >50% of hospitalizations in young infants [4]. RSV dominates the winter season, but its burden may soon change should maternal immunization strategies or RSV-specific monoclonal antibodies (mAbs) of prolonged half-life prevent severe disease. Human metapneumovirus often affects slightly older infants, extending the SWAB season into early spring [4]. Human rhinoviruses (hRVs) and human parainfluenza virus type 3 (hPIV3) dominate the fall and spring [4]. Interestingly, not all hRVs are the same, and severely ill hosts who are frequently “unmasked” by the pathogen include children with a specific at-risk background: premature infants with bronchopulmonary dysplasia, children with atopic backgrounds, and future asthmatics. hPIV3 and influenza viruses present, respectively, with more pneumonia or pharyngitis and fever. All these illnesses caused by different viruses prevail in slightly different age and risk groups and exhibit different genetic susceptibilities and varying cytokine profiles [4], but clinical presentations overlap sufficiently to cloud the diagnosis, making distinctions at bedside difficult if not impossible. Importantly, acute episodes can have markedly different long-term consequences. For instance, preventing severe acute RSV disease with a specific mAb lowers the incidence of recurrent wheezing until age 5 years, despite simultaneously increasing the absolute rate of infections with other viruses, such as hRVs [5, 6].

Furthermore, even RSV SWAB is pleomorphic in its clinical presentation and can manifest with significant differences in short- and long-term consequences for specific subgroups. In middle-class urban and suburban populations, infants with loss-of-function single-nucleotide polymorphisms in Asp299Gly and/or Thr399Ile (Toll-like receptor 4 [TLR4]^+/–^) experience exaggerated Th2 responses in the respiratory tract during RSV infection and are not protected by the administration of RSV-specific mAbs when premature [7]. In addition, infants with a TLR4^+/–^ genotype born at term experience an exorbitant approximately 90% hospitalization rate when visiting an emergency department with respiratory symptoms [7]. Children in Navajo and Apache reservations and those from indigenous peoples in Alaska are particularly susceptible to RSV, for reasons that remain unclear [8]. Their hospitalization rates significantly exceed those of other US children, but surprisingly also exceed those in low-income populations in the developing world. Furthermore, unlike in the studies from Europe and Japan, a high-affinity mAb against RSV failed to prevent long-term recurrent wheezing in healthy Native American infants, despite reducing the rate of severe acute RSV disease [9]. Other groups with increased susceptibility to RSV remain to be studied further, such as infants of asthmatic mothers or those with Down syndrome [4]. Preterm infants, presumptively due to reduced levels of forced expiratory flows, are also at greater risk for severe bronchiolitis and recurrent wheeze during the first year of life [4].

Times are changing. Bronchiolitis evidently is not a single disease, but a collective noun used to describe a set of clinical symptoms and features that arise through different pathophysiological mechanisms. Subtypes of bronchiolitis sharing similar observable characteristics are often labeled as phenotypes; SWAB “endotypes” should be defined on the basis of pathophysiological mechanisms [10]. “Endotypes” are subtypes of a condition with overlapping clinical symptoms, but each caused by a distinct underlying pathophysiological mechanism. In addition to the information derived from traditional hypothesis-driven studies, delineation of endotypes should benefit from advances in novel approaches to identify susceptibility genes for these ailments; next-generation sequencing technologies that enable single-cell RNA sequencing; and emerging fields of large-scale, data-rich biology. Noninvasive methods for measuring lung function may also be of value in defining SWAB endotypes. In these times of minimalistic approaches to pediatric practice, large collaborative prospective studies gathering detailed clinical information and laboratory samples should be fostered by governments and foundations, if we are to discriminate these disorders from each other.

One approach to “endotype” discovery, extensively used in asthma [10], uses data mining with various data-driven statistical and machine learning techniques to uncover patterns of clinical symptoms, different biomarkers, or “omics” data. This approach assumes that discovered patterns reflect pathophysiological mechanisms [10]. In the field of bronchiolitis, one such technique (latent class analysis) identified 3 severe bronchiolitis profiles [11]. Approximately half of infants were clustered in a subgroup resembling typical RSV bronchiolitis, a third experienced very severe disease, and the rest—at increased risk for subsequent recurrent wheezing—were most often infected with rhinoviruses and had higher eosinophil counts and cathelicidin levels [11]. Two other cohorts identified an additional profile of nonwheezing patients with milder illness. Recently, untargeted metabolomic analyses of urine in children with bronchiolitis discriminated those prone to recurrent wheezing as having a greater involvement of the citric acid cycle [12]. However, to be genuinely useful, any pattern recognition has to be coupled with both biological and clinical interpretation [10].

We can and should use responses to treatment for endotype discovery. Emergence of mAbs targeting specific cytokines or cytokine receptors potentially involved in pathogenesis of certain endotypes of SWAB may revolutionize the approach to disease diagnosis and alter disease severity, duration, and/or long-term consequences, but can also facilitate endotype discovery. For example, severe RSV disease in middle-class TLR4^+/–^ infants is associated with high levels of interleukin 4 in respiratory secretions [7], severe RSV bronchiolitis in the United Kingdom has been linked to interleukin 9 levels [13], and hRV life-threatening illness in low-income populations has been correlated with high levels of interleukin 13 [14]. Candidate interventions against these and other cytokines, today still costly, have been tested for other diseases [15].

Identification of “responders” to treatment with biologics may point to the pathways critically important for disease expression in different subgroups, thus enabling the discovery of SWAB endotypes. Other drugs not recommended for general administration today, such as β2 agonists, may find their niche once properly targeted. To be successful, we will have to find a way of bringing together information from population-based birth cohorts, studies in infants with manifest disease, and the results of RCTs and mechanistic studies, because none of these in isolation will provide sufficient information for disaggregation [10].

Much like asthma or fever, bronchiolitis is an umbrella term that harbors several diseases with similar clinical manifestations, likely both overlapping and unique mechanisms. Understanding the endotypes within SWAB will permit the move from the current symptom-based approach toward mechanism-based treatments, enabling targeted interventions to decrease the burden of illness on infants, their families, and society.

## Notes

***Acknowledgments.* **F. P. P. has received grants from Novavax and Janssen, and personal fees from Novavax, Medimmune, Pfizer, Merck, Bavarian Nordic, VirBio, Sanofi, GSK, Daiichi Sankyo, and ReViral. R. T. S. has received grants from Novavax, and personal fees from Novavax, Medimmune, Janssen, and Sanofi. A. C. has received personal fees from Novartis, Regeneron/Sanofi, Thermo Fisher Scientific, Boehringer Ingelheim, Novartis, and Philips.

***Financial support.* **This work was supported by the Bill & Melinda Gates Foundation (to F. P. P.).

***Potential conflicts of interest.* **All authors: No reported conflicts of interest. All authors have submitted the ICMJE Form for Disclosure of Potential Conflicts of Interest. Conflicts that the editors consider relevant to the content of the manuscript have been disclosed.
